# Modeling Working Memory in a Spiking Neuron Network Accompanied by Astrocytes

**DOI:** 10.3389/fncel.2021.631485

**Published:** 2021-03-31

**Authors:** Susanna Yu. Gordleeva, Yuliya A. Tsybina, Mikhail I. Krivonosov, Mikhail V. Ivanchenko, Alexey A. Zaikin, Victor B. Kazantsev, Alexander N. Gorban

**Affiliations:** ^1^Scientific and Educational Mathematical Center “Mathematics of Future Technology,” Lobachevsky State University of Nizhny Novgorod, Nizhny Novgorod, Russia; ^2^Neuroscience and Cognitive Technology Laboratory, Center for Technologies in Robotics and Mechatronics Components, Innopolis University, Innopolis, Russia; ^3^Center for Analysis of Complex Systems, Sechenov First Moscow State Medical University, Sechenov University, Moscow, Russia; ^4^Institute for Women's Health and Department of Mathematics, University College London, London, United Kingdom; ^5^Neuroscience Research Institute, Samara State Medical University, Samara, Russia; ^6^Department of Mathematics, University of Leicester, Leicester, United Kingdom

**Keywords:** spiking neural network, astrocyte, neuron-astrocyte interaction, working memory, delayed activity

## Abstract

We propose a novel biologically plausible computational model of working memory (WM) implemented by a spiking neuron network (SNN) interacting with a network of astrocytes. The SNN is modeled by synaptically coupled Izhikevich neurons with a non-specific architecture connection topology. Astrocytes generating calcium signals are connected by local gap junction diffusive couplings and interact with neurons via chemicals diffused in the extracellular space. Calcium elevations occur in response to the increased concentration of the neurotransmitter released by spiking neurons when a group of them fire coherently. In turn, gliotransmitters are released by activated astrocytes modulating the strength of the synaptic connections in the corresponding neuronal group. Input information is encoded as two-dimensional patterns of short applied current pulses stimulating neurons. The output is taken from frequencies of transient discharges of corresponding neurons. We show how a set of information patterns with quite significant overlapping areas can be uploaded into the neuron-astrocyte network and stored for several seconds. Information retrieval is organized by the application of a cue pattern representing one from the memory set distorted by noise. We found that successful retrieval with the level of the correlation between the recalled pattern and ideal pattern exceeding 90% is possible for the multi-item WM task. Having analyzed the dynamical mechanism of WM formation, we discovered that astrocytes operating at a time scale of a dozen of seconds can successfully store traces of neuronal activations corresponding to information patterns. In the retrieval stage, the astrocytic network selectively modulates synaptic connections in the SNN leading to successful recall. Information and dynamical characteristics of the proposed WM model agrees with classical concepts and other WM models.

## 1. Introduction

In neuroscience, the understanding of the functional role of astrocytes in the central nervous system (CNS) is still open to debate (Savtchouk and Volterra, 2018), but now there is accumulating evidence demonstrating the involvement of astrocytes in local synaptic plasticity and the coordination of network activity (Durkee and Araque, 2019), and as a result in information processing and memory encoding (Santello et al., 2019). Astrocytes sense synaptic activity and respond to it with the transient elevation of intracellular Ca^2+^ concentration (lasting from hundreds of a millisecond to dozens of seconds). Such Ca^2+^ signals in astrocytes have been observed in different brain regions and also in the cortex, appearing there in response to mechanic sensory stimulation (Wang X. et al., 2006; Takata et al., 2011; Stobart et al., 2018) and visual sensory stimulation (Schummers et al., 2008; Chen et al., 2012; Perea et al., 2014). Ca^2+^ activation can trigger the release of gliotransmitters from astrocytes, which in turn affect the dynamics of presynaptic and postsynaptic terminals resulting in modulations of synaptic transmission (Araque et al., 2014). The gliotransmitter-mediated synaptic modulation lasts from a dozen seconds (Jourdain et al., 2007; Perea et al., 2014) to a dozen minutes (Stellwagen and Malenka, 2006; Perea and Araque, 2007; Navarrete et al., 2012) contributing to both short- and long-term synaptic plasticity.

Obviously, there is a qualitative coincidence between the time scales of astrocyte-mediated synaptic modulation and the working memory (WM) timings during decision making. Based on this and the other following facts of astrocytes participation in neuronal signaling, we hypothesized that astrocytes may be involved in WM formation. In particular, recent *in vivo* studies have shown the participation of astrocytes in the synchronization of certain cortical network activities (Takata et al., 2011; Chen et al., 2012; Paukert et al., 2014; Perea et al., 2014), cognitive functions, and behaviors (Poskanzer and Yuste, 2016; Sardinha et al., 2017). Experimental evidence shows that astrocyte pathology in the medium prefrontal cortex (PFC) impairs WM and learning functions (Lima et al., 2014), increasing astrocyte density enhances short-term memory performance (Luca et al., 2020), and recognition memory performance and disruption of WM depend on gliotransmitter release from astrocytes in the hippocampus (Han et al., 2012; Robin et al., 2018). Despite these numerous experimental insights of the contribution of astrocytes to synaptic modulations in neuronal signaling, the possible role of astrocytes in information processing and learning is still a subject of discussion (Kanakov et al., 2019; Kastanenka et al., 2019).

Considering the significance of WM processes and the challenge of finding alternative mechanisms and experimental evidence of the astrocytic role in information processing in CNS, it is interesting to study astrocyte-induced modulation of synaptic transmission in WM organization. Specifically, we assume that the potentiation of excitatory synapses induced by Ca^2+^ elevation-mediated glutamate release from astrocytes (Fellin et al., 2004; Perea and Araque, 2007; Navarrete and Araque, 2008, 2010; Chen et al., 2012) plays an essential role in WM. To test this hypothesis, we developed a novel neuron-astrocyte network model for visual WM to reflect experimental data on the structure, connectivity, and neurophysiology of the neuron-astrocytic interaction in underlying cortical tissue. We focused on the implementation of a multi-item WM task in a delayed matching to sample (DMS) framework representing a classical neuropsychological paradigm (Miller et al., 1996). During the experiment, a test animal was given a sample stimulus, which it had to remember for several seconds until it could begin executing a certain task. For example, a stimulus was followed by a sequence of several test stimuli and the animal was rewarded for indicating when one of the test stimuli. matched the original sample. The DMS paradigm was previously studied using a recurrent neural network (Brunel and Wang, 2001; Amit, 2003; Amit et al., 2013; Fiebig and Lansner, 2016). The novelty of our model is that we associate memory with item-specific patterns of astrocyte-induced enhancement of excitatory synaptic transmission. We present a new case of how the biologically relevant neuron-astrocyte network model implements loading, storage, and cued retrieval of multiple items with significant overlapping. The memory items are encoded in neuronal populations in the form of discrete high-frequency bursts rather than persistent spiking.

In this paper, we review some related works (section 2), describe the proposed model and methods in detail (section 3), present the results (section 4), and finally, we conclude this work in section 5.

## 2. Related Work

The concept of WM implies the ability to temporarily store and process information in goal-directed behavior. WM is crucial in the generation of higher cognitive functions for both humans and other animals (Baddeley, 1986, 2012; Conway et al., 2003). In primates, visual WM has been studied in delay tasks, such as DMS, which require a memory to be held during a brief delay period lasting for several seconds (Miller et al., 1996). Recordings in the monkeys' PFCs during the delay task showed that some neurons displayed persistent and stimulus-specific delay-period activity (Fuster and Alexander, 1971; Funahashi et al., 1989; Shafi et al., 2007; Barak et al., 2010; Funahashi, 2017). Delay persistent activity is considered the neural correlate of WM (Goldman-Rakic, 1995; Constantinidis et al., 2018).

The classical theoretical memory models suggest that an information item can be stored with sustained neural activity which emerges via activation of stable activity patterns in the network (e.g., attractors) (Hopfield, 1982; Amit, 1995; Wang, 2001; Wimmer et al., 2014) recently reviewed by Zylberberg and Strowbridge (2017) and Chaudhuri and Fiete (2016). These WM models propose that the generation of persistent activity can be the result of an intrinsic property of the neurons [including the generation of the bistability mediated by the voltage-gated inward currents (Kass and Mintz, 2005) and Ca^2+^-triggered long-term changes in neuronal excitability (Fransén et al., 2006)] and can be induced by the connectivity within the neural circuit with feed-forward (Ganguli and Latham, 2009; Goldman, 2009) or recurrent architecture (Koulakov et al., 2002; Kilpatrick et al., 2013). In such models, memory recall is impossible from a silent inactive state. For many WM models of persistent activity based on recurrent connectivity, small deviations in the network structure destroy the persistence. Moreover, a spiking form of information storage is energetically unfavorable because of the high metabolic value of action potentials (Attwell and Laughlin, 2001).

In theoretical studies, a concept of oscillatory sub-cycles storing 7 ± 2 in oscillatory neuronal networks was proposed by Lisman and Idiart (1995). Other models employ oscillatory activity of spiking neuron networks after depolarization to memorize a set of information patterns at different phases of rhythmic oscillations (Klinshov and Nekorkin, 2008; Borisyuk et al., 2013).

Recently, the persistent activity hypothesis has been undergoing critical reviews (Lundqvist et al., 2018) based on the experimental findings in rodents and primates showing that the robust persistent activity does not last for the entire delay period, but rather sequential neuronal firing is observed during the delay period suggesting that the PFC neural network may support WM based on dynamically changing neuronal activity (Fujisawa et al., 2008; Lundqvist et al., 2016; Runyan et al., 2017; Park et al., 2019; Ozdemir et al., 2020). Despite the considerable progress that has been made in identifying the neurophysiological mechanisms contributing to WM in mammals (D'Esposito and Postle, 2015; Zylberberg and Strowbridge, 2017), the ongoing debate focuses on the generation mechanisms of the delay period activity that appears to underlie WM (Constantinidis et al., 2018; Sreenivasan and D'Esposito, 2019).

Currently, one of the recognized experimentally based hypotheses of the WM mechanism underlining the delay activity (not necessarily persistent) is the synaptic plasticity in the PFC (Tsodyks and Markram, 1997; Hempel et al., 2000; Wang Y. et al., 2006; Erickson et al., 2010). Synaptic plasticity implies a rapid regulation of the strengths of individual synapses in response to specific patterns of correlated synaptic activity and contributes to the activity-dependent refinement of neural circuitry. Following these findings, alternative synaptic-based WM models have been proposed (Mongillo et al., 2008; Barak and Tsodyks, 2014; Koutsikou et al., 2018; Manohar et al., 2019). In these models, memory items are stored by stimulus-specific patterns of synaptic facilitation in a neuronal circuit. Synaptic plasticity does not require neurons to show a persistent activity for the entire period of the memory task, which results in a robust and more metabolically efficient mechanism. Some synaptic WM models based on short-term non-associative synaptic facilitation (Mongillo et al., 2008; Lundqvist et al., 2011; Mi et al., 2017) allow for reading out and refreshing existing representations maintained in the synaptic structure. Others have proposed fast Hebbian activity-dependent synaptic plasticity (Sandberg et al., 2003; Fiebig and Lansner, 2016) for encoding and maintenance of novel associations.

Despite the numerous models describing the astrocytic impact on signaling in neuronal networks (see Oschmann et al., 2018 for a recent review) (Makovkin et al., 2020), there are few attempts to theoretically investigate the role of astrocyte-induced modulation of synaptic transmission in memory formation. Shen and Wilde (2007) demonstrate one of the first results of simulating the coupling of a Hopfield neural network, astrocytes, and cerebrovascular activity. Although this is not yet a true biophysical model, the results suggest that a modification of the synapse strengths allows the neuronal firing and the cerebrovascular flow to be compatible on a meso-scale; with astrocyte signaling added, limit cycles exist in the coupled networks. Tewari and Parpura (2013) and Wade et al. (2011) study how bidirectional coupling between astrocytes and neurons in small neuron-astrocyte ensembles mediates learning and dynamic coordination in the brain. A recent interesting theoretical study proposes a self-repairing spiking astrocyte-neural network combined with a novel learning rule based on the spike-timing-dependent plasticity and Bienenstock, Cooper, and Munro learning rule (Liu et al., 2019).

## 3. Materials and Methods

### 3.1. Neuron-Astrocyte Network Model

Even though the balance of inhibition and excitation was shown to play an important role in WM stabilization and can influence WM capacity (Barak and Tsodyks, 2014), we focus on the properties of astrocyte-induced modulation of excitatory synaptic transmission in the PFC. We take spiking neuronal network with dimension *W* × *H* consisting of synaptically coupled excitatory neurons formed by the Izhikevich model (Izhikevich, 2003). Neurons in the network are connected randomly with the connection length determined by the exponential distribution.

**Figure 1:**
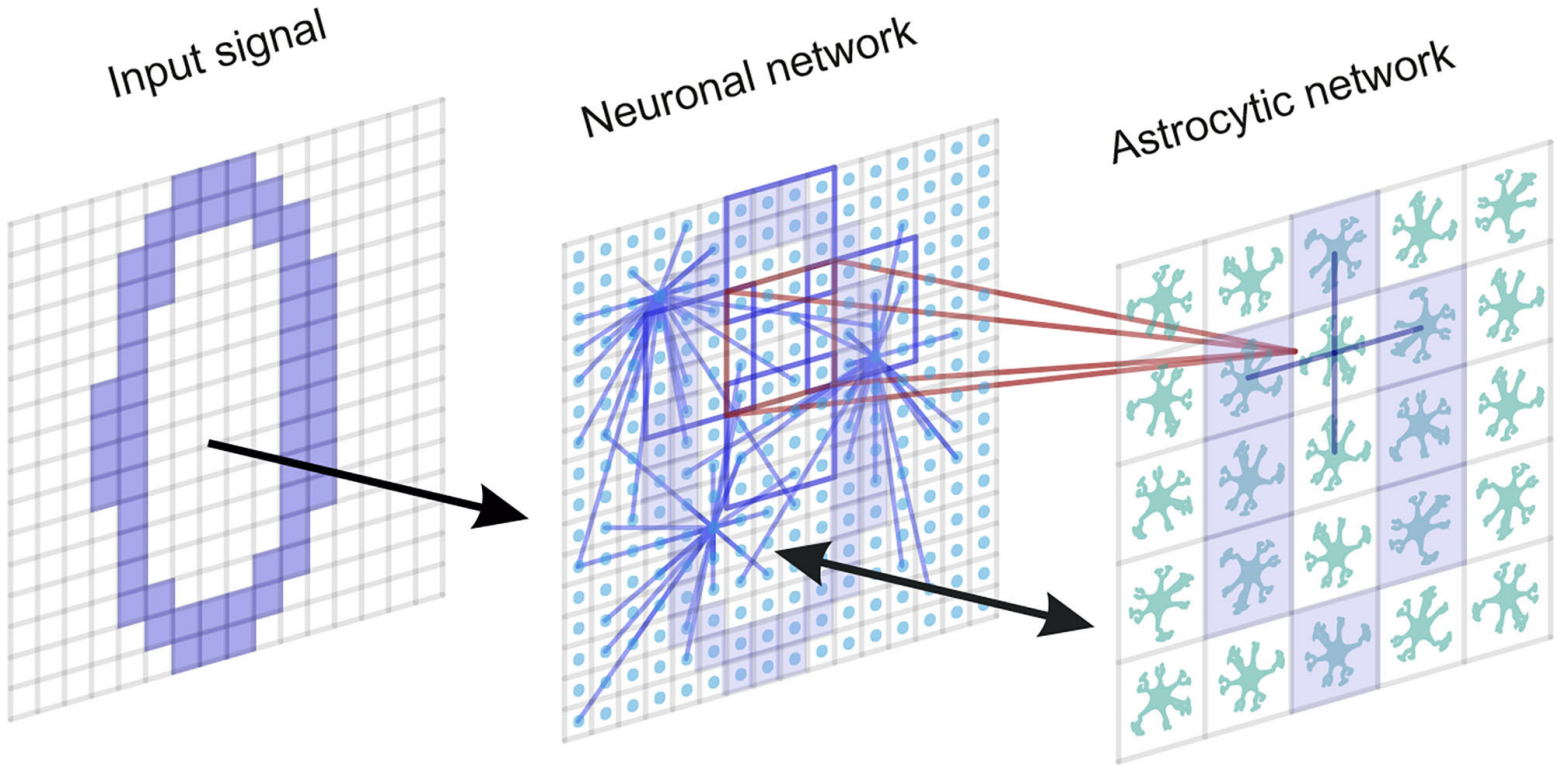
A neuron-astrocyte network topology. The neuron-astrocyte network consists of two interacting layers: a neural network layer and an astrocytic layer. The neuronal network with dimension *W* × *H* (79 × 79) consists of synaptically coupled excitatory neurons modeled by the Izhikevich neuron. Neurons in the network are connected randomly. The astrocytic network consists of diffusely connected astrocytes with dimension *M* × *N* (26 × 26). Blue lines show connections between elements in each layer. We consider the bidirectional interaction between the neuronal and astrocytic layers. Each astrocyte is interconnected with a neuronal ensemble of *N*_*a*_ = 16 neurons with dimensions 4 × 4 (red lines) with overlapping in one row (violet lines). The input signal is fed to the neural network.

It has been experimentally estimated that there is some overlap in the spatial territories occupied by individual astrocytes in the cortex (Halassa et al., 2007). An individual cortical astrocyte contacts on average 4-8 neuronal somata and 300–600 neuronal dendrites (Halassa et al., 2007). A cortical astrocyte has a “bushy” ramified structure in fine perisynaptic processes, which cover most of the neuronal membranes within their reach (Allen and Eroglu, 2017). This allows the astrocyte to integrate and coordinate a unique volume of synaptic activity. Following the experimental data, the astrocytic network compartment of our model is organized into a two-dimensional square lattice with only nearest-neighbor connectivity. Each astrocyte interacts with the neuronal ensemble of *N*_*a*_ neurons with some overlapping. We consider bidirectional communication between neuronal and astrocytic networks. The scheme of the network topology is shown in Figure 1.

Model equations are integrated using the Runge-Kutta fourth-order method with a fixed time step, Δ*t* = 0.1 ms. A detailed list of model parameters and values can be found in Table 1 (neural network model), Table 2 (astrocytic network parameters), Table 3 (neuron-astrocytic interaction parameters), and Table 4 (stimulation and recall testing). The code is available at: https://github.com/altergot/neuro-astro-network.

**Table 1:** Neural network parameters Izhikevich (2003), Kazantsev and Asatryan (2011).

**Parameter**	**Parameter description**	**Value**
*W* × *H*	Neural network grid size	79 × 79
*a*	Time scale of the recovery variable	0.1
*b*	Sensitivity of the recovery variable to the sub-threshold fluctuations of the membrane potential	0.2
*c*	After-spike reset value of the membrane potential	−65 mV
*d*	After-spike reset value of the recovery variable	2
η	Synaptic weight without astrocytic influence	0.025
*E*_syn_	Synaptic reversal potential for excitatory synapses	0 mV
*k*_syn_	Slope of the synaptic activation function	0.2 mV
*N*_out_	Number of output connections per each neuron	40
λ	Rate of the exponential distribution of synaptic connections distance	5

**Table 2:** Astrocytic network parameters Ullah et al. (2006).

**Parameter**	**Parameter description**	**Value**
*M* × *N*	Astrocytic network grid size	26 × 26
*c*_0_	Total Ca^2+^ in terms of cytosolic vol	2.0 μM
*c*_1_	(ER vol)/(cytosolic vol)	0.185
*v*_1_	Max Ca^2+^ channel flux	6 s^−1^
*v*_2_	Ca^2+^ leak flux constant	0.11 s^−1^
*v*_3_	Max Ca^2+^ uptake	2.2 μM s^−1^
*v*_6_	Maximum rate of activation-dependent calcium influx	0.2 μM s^−1^
*k*_1_	Rate constant of calcium extrusion	0.5 s^−1^
*k*_2_	Half-saturation constant for agonist-dependent calcium entry	1 μM
*k*_3_	Activation constant for ATP-Ca^2+^ pump	0.1 μM
*d*_1_	Dissociation constant for IP_3_	0.13 μM
*d*_2_	Dissociation constant for Ca^2+^ inhibition	1.049 μM
*d*_3_	Receptor dissociation constant for IP_3_	943.4 nM
*d*_5_	Ca^2+^ activation constant	82 nM
α		0.8
*v*_4_	Max rate of IP_3_ production	0.3 μM s^−1^
1/τ_*r*_	Rate constant for loss of IP_3_	0.14 s^−1^
${IP_{3}}^{*}$	Steady state concentration of IP_3_	0.16 μM
*k*_4_	Dissociation constant for Ca^2+^ stimulation of IP_3_ production	1.1 μM
*d*_*Ca*_	Ca^2+^ diffusion rate	0.05 s^−1^
*d*_*I*_*P*__3__	IP_3_ diffusion rate	0.1 s^−1^

**Table 3:** Neuron-astrocytic interaction parameters Gordleeva et al. (2012).

**Parameter**	**Parameter description**	**Value**
*N*_*a*_	Number of neurons interacting with one astrocyte	16, 4 × 4
α_glu_	Glutamate clearance constant	10 s^−1^
*k*_glu_	Efficacy of the glutamate release	600 μM s^−1^
*A*_*glu*_	Rate of IP_3_ production through glutamate	5 μM s^−1^
*t*_glu_	Duration of IP_3_ production through glutamate	60 ms
*G*_thr_	Threshold concentration of glutamate for IP_3_ production	0.7
*F*_act_	Fraction of synchronously spiking neurons required for the emergence of Ca^2+^ elevation	0.5
*F*_astro_	Fraction of synchronously spiking neurons required for the emergence of astrocytic modulation of synaptic transmission	0.375
$\nu_{Ca}^{*}$	Strength of the astrocyte-induced modulation of synaptic weight	0.5
${\left[Ca^{2+}\right]}_{\text{thr}}$	Threshold concentration of Ca^2+^ for the astrocytic modulation of the synapse	0.15 μM
τ_astro_	Duration of the astrocyte-induced modulation of the synapse	250 ms

**Table 4:** Stimulation protocol and recall testing parameters.

**Parameter**	**Parameter description**	**Value**
*f*_*bg*_	Background activity rate	1.5 Hz
*A*_stim_	Stimulation amplitude	10 μA
*t*_stim_	Stimulation duration	200 ms
	Noise level in sample	5%
*A*_test_	Cue stimulation amplitude	8 μA
*t*_test_	Cue stimulation length	150 ms
	Noise level in cue	20%

### 3.2. Neuronal Network

There are many available biophysical models of neuronal membrane potential dynamics (Morris and Lecar, 1981; Hodgkin and Huxley, 1990; Xu et al., 2020a,b; Zhang et al., 2020). For our purpose we chose the Izhikevich model as it is quite functional and computationally effective for network simulations (Izhikevich, 2003):

(1) $$\begin{array}{l} \begin{array}{l} \frac{dV^{\left(i,j\right)}}{dt}=0.04V^{{\left(i,j\right)}^{\left(2\right)}}+5V^{\left(i,j\right)}-U^{\left(i,j\right)}+140+I_{\text{app}}^{\left(i,j\right)}+I_{\text{syn}}^{\left(i,j\right)};\\ \frac{dU^{\left(i,j\right)}}{dt}=a\left(bV^{\left(i,j\right)}-U^{\left(i,j\right)}\right); \end{array} \end{array}$$

with the auxiliary after-spike resetting

(2) $$\begin{array}{l} \text{if }V^{\left(i,j\right)}\geq \text{30 mV, then}\{\begin{array}{l} V^{\left(i,j\right)}\leftarrow c\\ U^{\left(i,j\right)}\leftarrow U^{\left(i,j\right)}+d, \end{array} \end{array}$$

where the superscripts (*i* = 1, …, 79, *j* = 1, …, 79) correspond to a neuronal index, the transmembrane potential *V* is given in mV and time *t* in ms. The applied currents $I_{\text{app}}^{\left(i,j\right)}$ simulate the input signal. Neurons receive a number of synaptic currents from other presynaptic neurons in the network, $N_{in}^{\left(i,j\right)}$, which are summed at the membrane according to the following equation (Kazantsev and Asatryan, 2011; Esir et al., 2018):

(3) $$\begin{array}{l} I_{\text{syn}}^{\left(i,j\right)}=\overset{N_{in}^{\left(i,j\right)}}{\underset{k=1}{\sum }}\frac{g_{\text{syn}}^{\left(i,j\right)}\left(E_{\text{syn}}-V^{\left(i,j\right)}\right)}{1+\exp\left(\frac{-V_{\text{pre}}^{k}}{k_{\text{syn}}}\right)}; \end{array}$$

Parameter $g_{\text{syn}}^{\left(i,j\right)}$ describes the synaptic weight, $g_{\text{syn}}^{\left(i,j\right)}=\eta +\nu_{Ca}^{\left(m,n\right)}$. The astrocyte (*m, n*) modulates the synaptic currents of the neuron (*i, j*). The variable ν_*Ca*_ introduces the astrocyte-induced modulation of synaptic strength and will be discussed below. In this study, we concentrate exclusively on the role of astrocyte-induced modulation of synaptic transmission and did not include mechanisms of synaptic plasticity in the model. The synaptic reversal potential for excitatory synapses is taken with *E*_syn_ = 0. *V*_pre_ denotes the membrane potential of the presynaptic neuron. For simplicity, we neglect the axonal and synaptic delays.

The architecture of synaptic connections between neurons is non-specific (random) with the following parameters. The number of output connections per each neuron is fixed at *N*_*out*_ = 40. Each neuron innervates *N*_*out*_ local postsynaptic targets, which are randomly chosen in polar coordinates. The distances between neurons *r* are determined according to the exponential distribution *f*_*R*_(*r*) and the angles ϕ are chosen from the uniform distribution in the range [0; 2π]:

(4) $$\begin{array}{l} f_{R}\left(r\right)=\{\begin{array}{l} 1/\lambda \exp\left(-\left(1/\lambda \right)r\right),r\geq 0,\\ 0,r<0. \end{array} \end{array}$$

In this way, the coordinates of postsynaptic neurons are computed as follows:

(5) $$\begin{array}{l} x_{post}=\left\lceil x_{pre}+r\cos\left(\phi \right)\right\rceil ,y_{post}=\left\lceil y_{pre}+r\sin\left(\phi \right)\right\rceil , \end{array}$$

where *x*_*pre*_, *y*_*pre*_ denote the coordinates of the presynaptic neuron, *x*_*post*_, *y*_*post*_ are coordinates of the postsynaptic neurons. Coordinates are picked repeatedly in case of a duplicated connection.

### 3.3. Astrocytic Network

In the model, we try to implement the biologically plausible organization of the astrocytic network and neuron-astrocyte interaction. The astrocytic network is configured in the form of a two-dimensional square lattice with dimension *M* × *N*. Cortical astrocytes are coupled via Cx43 gap junctions mostly permeable to inositol 1,4,5-trisphosphate (IP_3_) (Yamamoto et al., 1990; Nagy and Rash, 2000). Hence, in the model, we consider local diffusive coupling. Besides, each astrocyte is interconnected with a neuronal ensemble of *N*_*a*_ neurons. It was experimentally shown that the sensory stimulation evokes fast intracellular Ca^2+^ signals in fine processes of cortical astrocytes in response to local synaptic activity in the neuronal circuit (Wang X. et al., 2006; Takata et al., 2011; Stobart et al., 2018). Multiple rapid spatially restricted Ca^2+^ events in the astrocytic process are induced by intense neuronal firing. Local events are spatially and temporally integrated by the astrocytic cell, which results in a global long lasting Ca^2+^ event. In turn, this event induces the release of gliotransmitters affecting synaptic transmission in the local territory of individual astrocytes (Bekar et al., 2008; Henneberger et al., 2010; Araque et al., 2014). For simplicity, we did not model the detailed process of spatial-temporal integration of the rapid Ca^2+^ signals in the morphological structure of astrocytes modeled earlier by Gordleeva et al. (2018, 2019) and Wu et al. (2018). Here we employ a mean-field approach to describe the emergence of a global Ca^2+^ signal and its impact on the synchronization of neuronal ensemble controlled by a certain astrocyte.

As pyramidal neurons generate the spike, glutamate is released from the presynaptic terminal into the synaptic cleft (Figures 3B,D). The amount of glutamate, *G*, that was diffused from the synaptic cleft and reached the astrocytic process can be described by the following equation (Gordleeva et al., 2012; Pankratova et al., 2019):

(6) $$\begin{array}{l} \frac{dG^{\left(i,j\right)}}{dt}=-\alpha_{\text{glu}}G^{\left(i,j\right)}+k_{\text{glu}}\Theta \left(V^{\left(i,j\right)}-30mV\right), \end{array}$$

here α_glu_ is the glutamate clearance constant, *k*_glu_ is the efficacy of the release, Θ denotes the Heaviside step function, and *V*^(*i, j*)^ is the membrane potential of the corresponding presynaptic neuron (*i, j*). Binding of glutamate to metabotropic glutamate receptors (mGluR) on the astrocytic membrane, which is located close to the synapse, triggers the production of IP_3_ in the astrocytes (Figure 3E). We use the approaches from earlier studies to describe the dynamics of the intracellular concentration of IP_3_ in astrocytes (Nadkarni and Jung, 2003; Ullah et al., 2006):

(7) $$\begin{array}{ll} \frac{dIP_{3}^{\left(m,n\right)}}{dt}=\frac{IP_{3}^{*}-IP_{3}^{\left(m,n\right)}}{\tau_{IP3}} & +J_{\text{PLC}\delta }^{\left(m,n\right)}+J_{\text{glu}}^{\left(m,n\right)}+{\text{diff}}_{IP3}^{\left(m,n\right)}, \end{array}$$

with *m* = 1, …, 26, *n* = 1, …, 26. Parameter $IP_{3}^{*}$ denotes the steady state concentration of the IP_3_ and *J*_PLCδ_ describes the IP_3_ production by phospholipase Cδ (PLCδ) (Ullah et al., 2006):

(8) $$\begin{array}{l} J_{\text{PLC}\delta }=\frac{v_{4}\left(\left[Ca^{2+}\right]+\left(1-\alpha \right)k_{4}\right)}{\left[Ca^{2+}\right]+k_{4}} \end{array}$$

The variable *J*_glu_ describes the glutamate-induced production of the IP_3_ in response to neuronal activity and is modeled as a rectangular-shaped pulse with amplitude *A*_glu_ μM and duration *t*_glu_ ms:

(9) $$\begin{array}{l} J_{\text{glu}}=\{\begin{array}{ll} A_{\text{glu}},\; & \text{if }t_{0}<t\leq t_{0}+t_{\text{glu}},\\ 0,\; & \text{otherwise}; \end{array} \end{array}$$

here *t*_0_ denotes the periods when the total level of glutamate in all synapses associated with this astrocyte reaches a threshold:

(10) $$\begin{array}{l} \left(\frac{1}{N_{a}}\underset{\left(i,j\right)\in N_{a}}{\sum }\left[G^{\left(i,j\right)}>G_{\text{thr}}\right]\right)>F_{act}, \end{array}$$

here we use the parameter *G*_thr_ = 0.7. [*x*] denotes the Iverson bracket. *F*_act_ is the fraction of synchronously spiking neurons of the neuronal ensemble corresponding to the astrocyte. For the emergence of the calcium, elevation *F*_act_ = 0.5 is required. In other words, according to the experimental data (Bindocci et al., 2017), activation of the production term, *J*_glu_, which results in the generation of a calcium signal in the astrocyte, can be induced only when correlated activity in the neuronal ensemble interacting with the astrocyte reaches a sufficient level of coherence.

Increase of IP_3_ concentration in the astrocytes induces the release of Ca^2+^ from internal stores, mostly from the endoplasmic reticulum (ER), to cytosol. For a simplified description of the biophysical mechanism underlying the calcium dynamics in astrocytes, we use the Ullah model (Ullah et al., 2006). Changes of the intracellular Ca^2+^ concentration, [*Ca*^2+^], are described by the following equations:

(11) $$\begin{array}{l} \frac{d{\left[Ca^{2+}\right]}^{\left(m,n\right)}}{dt}=J_{\text{ER}}^{\left(m,n\right)}-J_{\text{pump}}^{\left(m,n\right)}+J_{\text{leak}}^{\left(m,n\right)}+J_{\text{in}}^{\left(m,n\right)}-J_{\text{out}}^{\left(m,n\right)}\\ \;+{\text{diff}}_{Ca}^{\left(m,n\right)};\\ \frac{dh^{\left(m,n\right)}}{dt}=a_{2}\left(d_{2}\frac{IP_{3}^{\left(m,n\right)}+d_{1}}{IP_{3}^{\left(m,n\right)}+d_{3}}\left(1-h^{m,n}\right)-{\left[Ca^{2+}\right]}^{\left(m,n\right)}h^{\left(m,n\right)}\right); \end{array}$$

where *h* is the fraction of the activated IP_3_ receptors (IP_3_Rs) on the ER surface. Flux *J*_ER_ is Ca^2+^ flux from the ER to the cytosol through IP_3_Rs, *J*_pump_ is the Ca^2+^ flux pumped back into ER via the sarco/ER Ca^2+^-ATPase (SERCA), and *J*_leak_ is the leakage flux from the ER to the cytosol. Fluxes *J*_in_ and *J*_out_ describe the calcium exchange with extracellular space. The fluxes are expressed as follows:

(12) $$\begin{array}{l} J_{\text{ER}}=c_{1}v_{1}{\left[Ca^{2+}\right]}^{3}h^{3}IP_{3}^{3}\frac{\left(c_{0}/c_{1}-(1+1/c_{1})\left[Ca^{2+}\right]\right)}{((IP_{3}+d_{1}){\left(\left[Ca^{2+}\right]+d_{5}\right)}^{3}};\\ J_{\text{pump}}=\frac{v_{3}{\left[Ca^{2+}\right]}^{2}}{k_{3}^{2}+{\left[Ca^{2+}\right]}^{2}};\\ J_{\text{leak}}=c_{1}v_{2}(c_{0}/c_{1}-(1+1/c_{1})\left[Ca^{2+}\right]);\\ J_{\text{in}}=\frac{v_{6}IP_{3}^{2}}{k_{2}^{2}+IP_{3}^{2}};\\ J_{\text{out}}=k_{1}\left[Ca^{2+}\right]; \end{array}$$

Biophysical meaning of all parameters in Equations (7), (8), (11), (12) and their experimentally determined values can be found in Li and Rinzel (1994), Ullah et al. (2006) and Table 2.

Cortical astrocytes are coupled by Cx43 gap junctions (Yamamoto et al., 1990; Nagy and Rash, 2000; Nimmerjahn et al., 2004). Thus, the diffusion of active chemicals becomes possible between the neighboring astrocytes. Currents diff_*Ca*_ and diff_*I*_*P*__3__ describe the diffusion of Ca^2+^ ions and IP_3_ molecules via gap junctions between the astrocytes in the network and can be expressed as follows:

(13) $$\begin{array}{l} \begin{array}{l} {\text{diff}}_{Ca}^{\left(m,n\right)}=d_{Ca}{\left(\Delta \left[Ca^{2+}\right]\right)}^{\left(m,n\right)};\\ {\text{diff}}_{IP3}^{\left(m,n\right)}=d_{IP3}{\left(\Delta IP_{3}\right)}^{\left(m,n\right)}; \end{array} \end{array}$$

where parameters *d*_*Ca*_ and *d*_*I*_*P*__3__ describe the Ca^2+^ and IP_3_ diffusion rates, respectively. Following experimental data, we assume that Cx43 is less permeable to Ca^2+^ than to IP_3_. We consider that each astrocyte is diffusively coupled with only four nearest-neighbors. (Δ[*Ca*^2+^])^(*m, n*)^ and ${\left(\Delta IP_{3}\right)}^{\left(m,n\right)}$ are the discrete Laplace operators:

(14) $$\begin{array}{l} {\left(\Delta \left[Ca^{2+}\right]\right)}^{\left(m,n\right)}=({\left[Ca^{2+}\right]}^{\left(m+1,n\right)}+{\left[Ca^{2+}\right]}^{\left(m-1,n\right)}\\ \;+{\left[Ca^{2+}\right]}^{\left(m,n+1\right)}+{\left[Ca^{2+}\right]}^{\left(m,n-1\right)}\\ \;\;-4{\left[Ca^{2+}\right]}^{\left(m,n\right)}). \end{array}$$

Equations (7)–(9), (11)–(13) predict that the synchronized activity in the neuronal ensemble trigger astrocytic Ca^2+^ signals, and in the absence of neuronal stimulus in the astrocytic network, steady state Ca^2+^ concentration is maintained.

Next, we account for the effect of the enhancement of excitatory synaptic transmission through the action of the glutamate released from astrocytes. We consider that the astrocytic glutamate-induced potentiation of the synapse consists in NMDAR-dependent postsynaptic slow inward currents (SICs) generation (Fellin et al., 2004; Chen et al., 2012) and mGluR-dependent heterosynaptic facilitation of presynaptic glutamate release (Perea and Araque, 2007; Navarrete and Araque, 2008, 2010). In the model, we propose that global events of Ca^2+^ elevation in astrocytes result in glutamate release, which can modulate the synaptic strength of all synapses corresponding to the morphological territory of a given astrocyte. For simplicity, the relationship between the astrocyte Ca^2+^ concentration and synaptic weight of the affected synapses *g*_syn_, is described as follows:

(15) $$\begin{array}{l} \begin{array}{l} g_{\text{syn}}=\eta +\nu_{Ca},\\ \nu_{Ca}=\nu_{Ca}^{*}\Theta \left({\left[Ca^{2+}\right]}^{\left(m,n\right)}-{\left[Ca^{2+}\right]}_{\text{thr}}\right), \end{array} \end{array}$$

where the parameter $\nu_{Ca}^{*}$ denotes the strength of the astrocyte-induced modulation of the synaptic weight and Θ(*x*) is the Heaviside step-function. The feedback from the astrocytes to the neurons is activated when the astrocytic Ca^2+^ concentration is larger than ${\left[Ca^{2+}\right]}_{\text{thr}}$ and the fraction of synchronously spiking neurons of the neuronal ensemble corresponding to the astrocyte *F*_astro_ during the time period of τ_syn_ = 10 ms. According to the experimental data on the kinetics of NMDAr-dependent SICs that is evoked by glutamate released from astrocytes (Fellin et al., 2004), the duration of the astrocyte-induced facilitation of synaptic transmission is fixed and is equal to τ_astro_ = 250 ms.

### 3.4. Stimulation Protocol

The term *I*_*app*_ in Equation (1) represents specific and non-specific external inputs. A non-specific noisy input simulates input signals from networks of other brain areas and is applied continuously to all neurons in the form of independent Poisson pulse trains of a certain rate, *f*_bg_, with amplitudes randomly and uniformly distributed in the interval [−10, 10] μA. This input evokes a background network state with low-rate spontaneous spiking.

**Figure 2:**
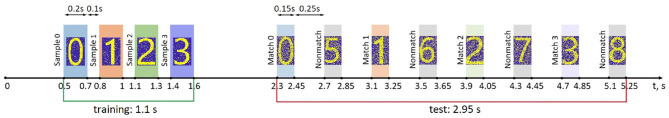
Time course of a training and test protocol in the multi-item WM paradigm used. During training the samples were loaded sequentially by applying external inputs of 200 ms durations with 100 ms inter-item intervals. After a 700 ms training stimulus was applied, we tested the maintenance of the memory by applying matching and non-matching items of 150 ms durations and 250 ms inter-item intervals.

Specific input contains training samples in the form of binary spatial patterns. The patterns represent different spatial distributions relative to background state with non-specific input only. The average size of a sample is 1,078 neurons (18% of the network) stimulated by the specific input, with an average 35.2% overlapping in the population. For a visual representation of samples, we take binary images of numerals (0,1,2,3,4,..) with size *W* × *H* pixels, where each pixel corresponds to a neuron in the neuronal layer. Neurons corresponding to the shape of the numerals receive a rectangular excitatory pulse with length *t*_stim_ and amplitude *A*_stim_. The shape of each sample was spatially distorted by 5% random noise such as “salt and pepper noise.” Then transient inputs were applied to simulate the nonmatching test items and the cue (length *t*_test_, and amplitude *A*_test_). In the cued recall for simulating the loss in saliency, we applied a shorter input with lower amplitude and a higher level (20%) of random noise. Figure 2 illustrates the time course of a training and test protocol used in the WM paradigm.

### 3.5. Memory Performance Metrics

To measure the memory performance of the system we calculate the correlation of a recalled pattern with the ideal item in the following way:

(16) $$\begin{array}{l} \begin{array}{l} M_{ij}\left(t\right)=I\left[\left(\overset{t}{\underset{k=t-w}{\sum }}I\left[V_{ij}\left(k\right)>thr\right]\right)>0\right],\\ C\left(t\right)=\frac{1}{2}\left(\frac{1}{\left|P\right|}\underset{\left(i,j\right)\in P}{\sum }M_{ij}\left(t\right)+\frac{1}{W\cdot H-|P|}\underset{\left(i,j\right)\notin P}{\sum }\left(1-M_{ij}\left(t\right)\right)\right),\\ C_{P}=\frac{1}{\left|T_{P}\right|}\underset{t\in T_{P}}{\max}C\left(t\right); \end{array} \end{array}$$

here *w* = 10 frames = 1 ms, *P*—a set of pixels belonging ideal pattern, *W, H*—network dimensions, *thr*—spike threshold, *I*—indicator function, and *T*_*P*_—a set of frames in the tracking range of pattern *P*. In a sense, this correlation metric can be associated with 1 − *d* averaged between the pattern and background, where *d* is the Hamming distance.

## 4. Results

Let us show how the neuron-astrocyte network model exhibits memory formation. First, we will consider a simple single-item memory task illustrating information loading, storage, and retrieval. Next, we will demonstrate how the network can be successfully trained to memorize and recall several patterns with significant overlaps. Finally, we will analyze model performance metrics, capacity, and characteristics of pattern remembering on different parameters.

### 4.1. Single-Item WM

**Figure 3:**
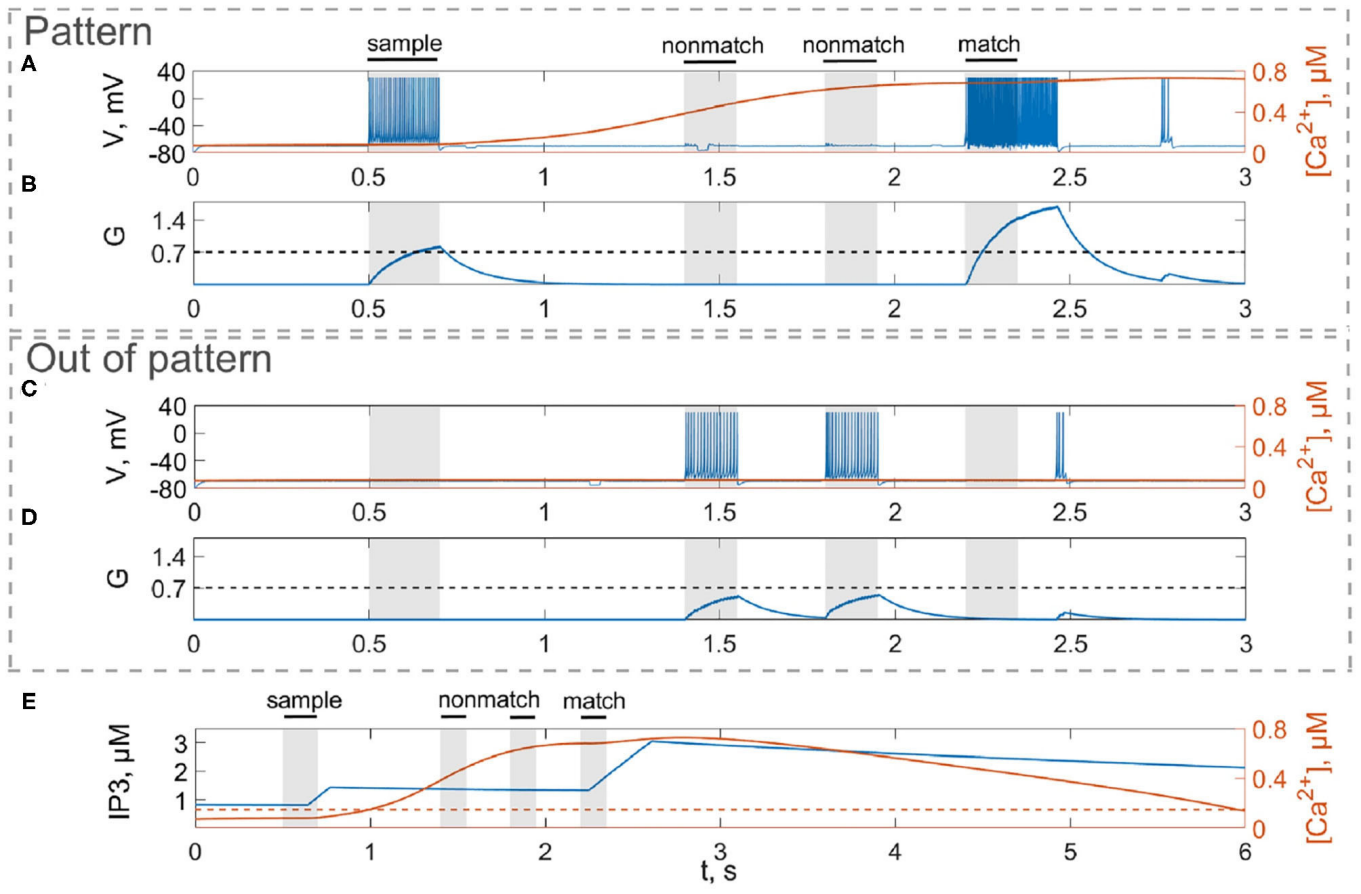
Model of neuron-astrocyte interaction. **(A)** Spike train and **(B)** concentration of the neurotransmitter, *G*(*t*), of the stimulus-specific neuron. **(C,D)** Same as in **(A,B)** but for an unspecific neuron. **(E)** Intracellular concentration of Ca^2+^ and IP_3_ in a stimulus-specific astrocyte. Black bars at the top indicate periods when each of the stimuli (training stimulus—sample, nonmatching test items—nonmatch, test cue—match) were presented. In response to the presynaptic spike train **(A,C)**, the neurotransmitter, glutamate, *G* releases **(B,D)** into extracellular space and the concentration of IP_3_ increases in the astrocyte (**E**, blue line) inducing the elevation of intracellular Ca^2+^ (**E**, red line).

**Figure 4:**
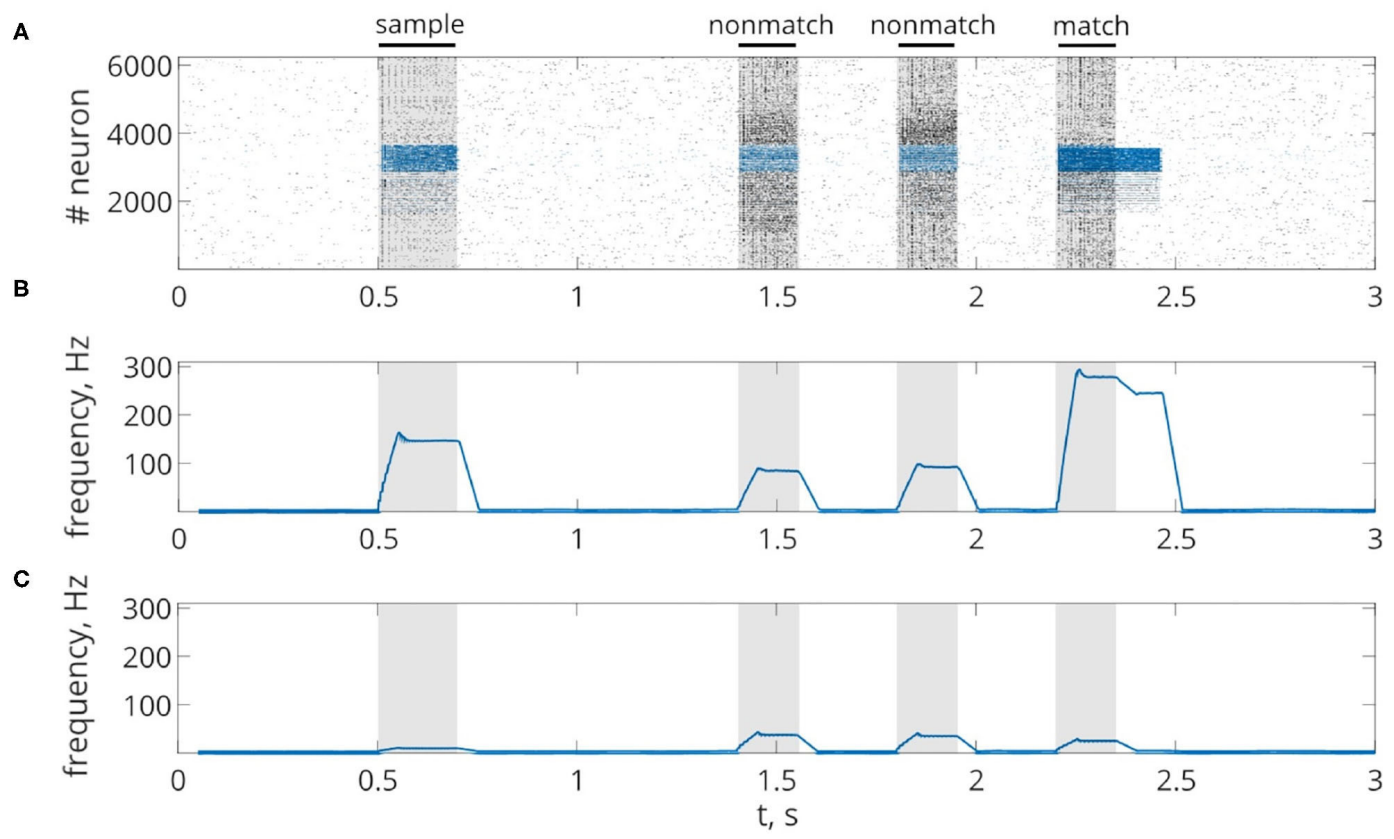
Delayed matching to sample WM task. **(A)** One trial of the task simulated in the network. Spike raster of the neuronal network showing sample-selective delay activity. Neurons belonging to the stimulus-specific population are indicated by blue color. Black bars at the top indicate periods when each of the stimuli (training stimulus—sample, nonmatching test items—non-match, test cue—match) were presented. **(B,C)** The averaged firing rate of the stimulus-specific and unspecific neurons over time, respectively (20 ms bins).

First, we will test the neuron-astrocyte network in the most common experimental paradigm of WM studies—the DMS task. This task requires a single item to be held in memory during a brief delay period. Before specific stimulation, the neural network demonstrates irregular, low-rate background activity (see activity beginning in Figure 4). At the 500 ms mark, we load an item by applying transient external input to the corresponding neuronal population for 200 ms (Figures 3A, 4). During training, each astrocyte tracks the activity of the neuronal subnetwork associated with it. As soon as the extracellular concentration of glutamate (Figures 3B,D) and correlated firing in neurons achieve a certain level, which satisfies the condition Equation (10), Ca^2+^ concentration in matching astrocytes elevates (Figure 3E). In accordance with the experimental data (Bindocci et al., 2017), we tuned the model parameters in such a way that the onset of calcium elevation in the astrocytes induced by synchronous neuronal discharge had a delay of ≤ 2 s. Following the increased firing in the stimulus-specific part of the neuronal network upon reaching a threshold of 0.15 μM, the astrocytes release gliotransmitters modulating the synaptic strengths in corresponding locations (Figure 3). The calcium pulse in astrocytes lasts for several seconds. Its duration determines the length of the delay interval in the DMS task, during which the item is maintained in the memory. After the training stimulus ends, we test maintenance of the single-item memory by applying two non-matching items and cue item with *t*_test_ durations and 250 ms inter-item intervals (Figures 3, 4). Because the astrocytic feedback also depends on the activity of the neuronal subnetwork, the model responds differently to the applied items. A short presentation of the cue to the neural network evokes the astrocytic-induced increase in the synaptic strength between stimulus-specific neurons and results in a local spatial synchronization in the whole stimulus-specific neuronal population (see Figure 3A in comparison to Figure 3C). Similar to experimental data (Miller et al., 1996), delay activity in our model is sample-selective. We observe that the pattern-specific firing rate in the neuronal network increases and is equal to 270 Hz in comparison with the response to a non-specific stimulus (80 Hz) (Figure 4B). Such a high frequency is determined by the choice of a fast-spiking neuron model (Izhikevich, 2003). The firing rates in simulations with a regular spiking neuron model (Izhikevich, 2003) are almost 10 times lower: 30 Hz for stimulus-specific and 4.5 Hz for non-specific stimulus. The elevation of the frequency in the stimulus-specific neuronal population can continue after the end of the cue, which is determined by the duration of the astrocyte-induced enhancement of the synaptic weight.

**Figure 5:**
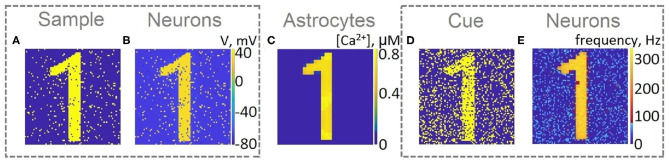
Snapshots of the training **(A–C)** and testing **(D,E)** of the neuron-astrocyte network in the single-item working memory task. **(A)** Training sample. **(B)** Response of the neuronal network to the sample. The values of the membrane potentials are shown. **(C)** Intracellular Ca^2+^ concentrations in the astrocytic layer. **(D)** Testing item with 20% salt and pepper noise. **(E)** Cued recall in the neuronal network. The averaged firing rate on the test time interval for each neuron is shown.

For a visual representation of memory formation, we follow the space-time distribution of sample-selective delay activity. Figure 5 illustrates the spatial distribution of activity in neuronal and astrocytic layers at the different moments of training and cued recall for the same single-item memory task as presented in Figure 4. Training induces the emergence of synchronized calcium activity of spatially clustered astrocytes (Figure 5C). Note that locally synchronized astrocytes have been found in the neocortex and hippocampus *in situ* and *in vivo* (Takata and Hirase, 2008; Sasaki et al., 2011). Such calcium activity correlated in time and space can lead to spatial-temporal synchronization in the neuronal network (Araque et al., 2014). This mechanism of neuron-astrocyte network interaction underlies the sample selectivity and pattern retrieval in the model. Note that 20 % of noisy cue items (Figure 5D) can be identified and cleared from noise by the neuronal network due to the astrocyte-induced feedback (Figure 5E). In other words, the spiking neuronal network accompanied by astrocytes can filter a cue pattern distorted by noise. Video of the single-item memory encoding and cued recall in the neuron-astrocyte network can be found in the Supplementary Material.

### 4.2. Multi-Item WM

**Figure 6:**
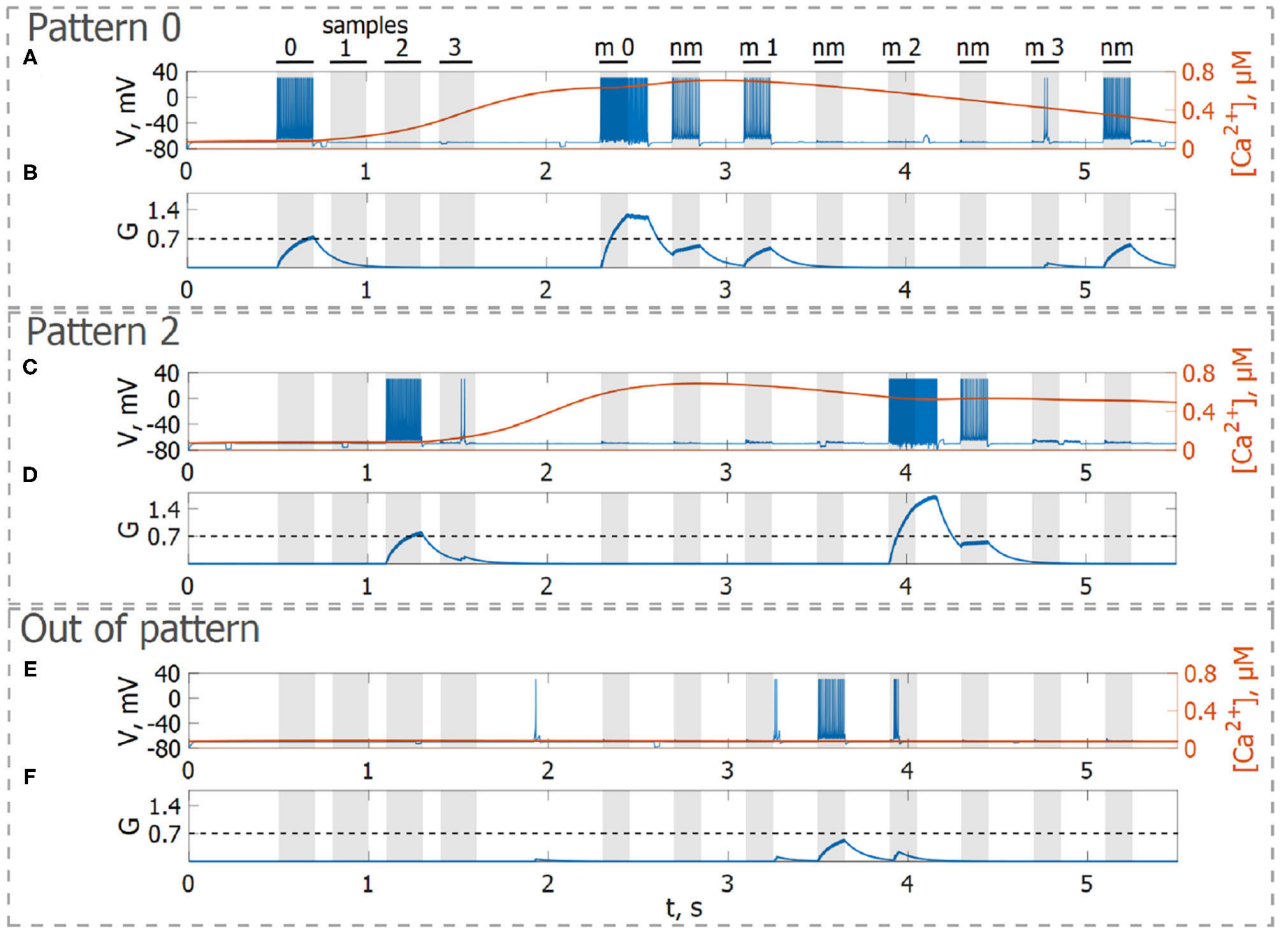
Neuron-astrocyte network simulation with four loaded memory items. **(A,C,E)** Spike train and **(B,D,F)** concentration of the neurotransmitter, *G*(*t*), of three neurons belonging to different stimulus-specific populations. **(A,B)** Stimulus-specific neuron to sample 0. **(C,D)** Stimulus-specific neuron to sample 2. **(E,F)** Neuron unspecific to all samples. Black bars at the top indicate periods when each of the stimuli (training stimulus—sample, non-matching test items—nm, match cue—m) were presented.

**Figure 7:**
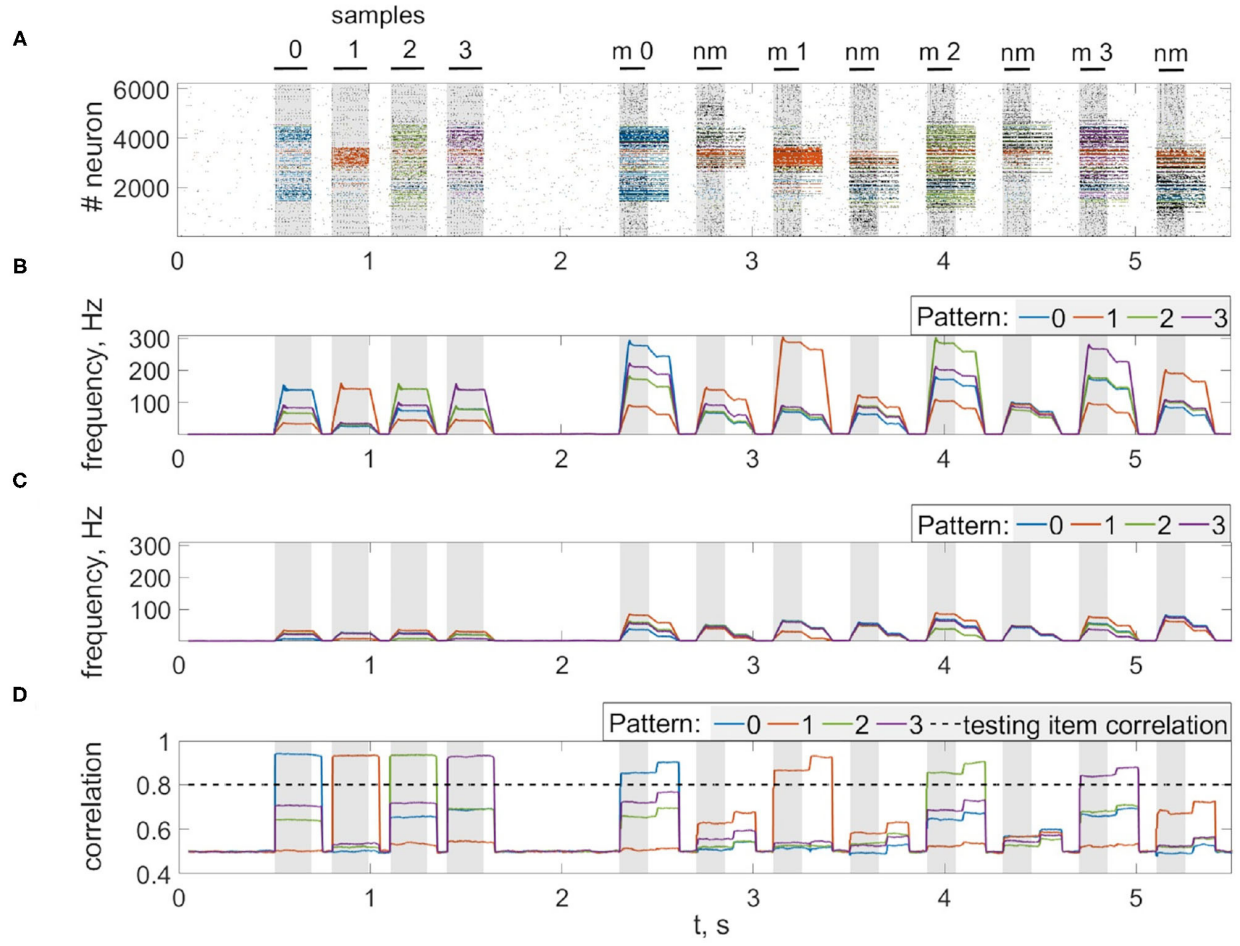
Multi-item WM in the neuron-astrocyte network. **(A)** Spike raster of the neuronal network with four training patterns. Neurons are colored according to their pattern selectivity. Pattern overlapping in neuronal populations is 35.2% on average for four patterns. Black bars indicate periods when each of the stimuli were presented. **(B,C)** The averaged firing rate of the stimulus-specific and unspecific neurons over time, respectively (20 ms bins). **(D)** Correlation of filtered items. The different colors correspond to the correlations with different ideal samples. The dotted line shows the correlation of the testing item (for 20% noise level in test). Black bars at the top indicate periods when each of the stimuli (training stimulus—sample, non-matching test items—nm, match cue—m) were presented.

**Figure 8:**
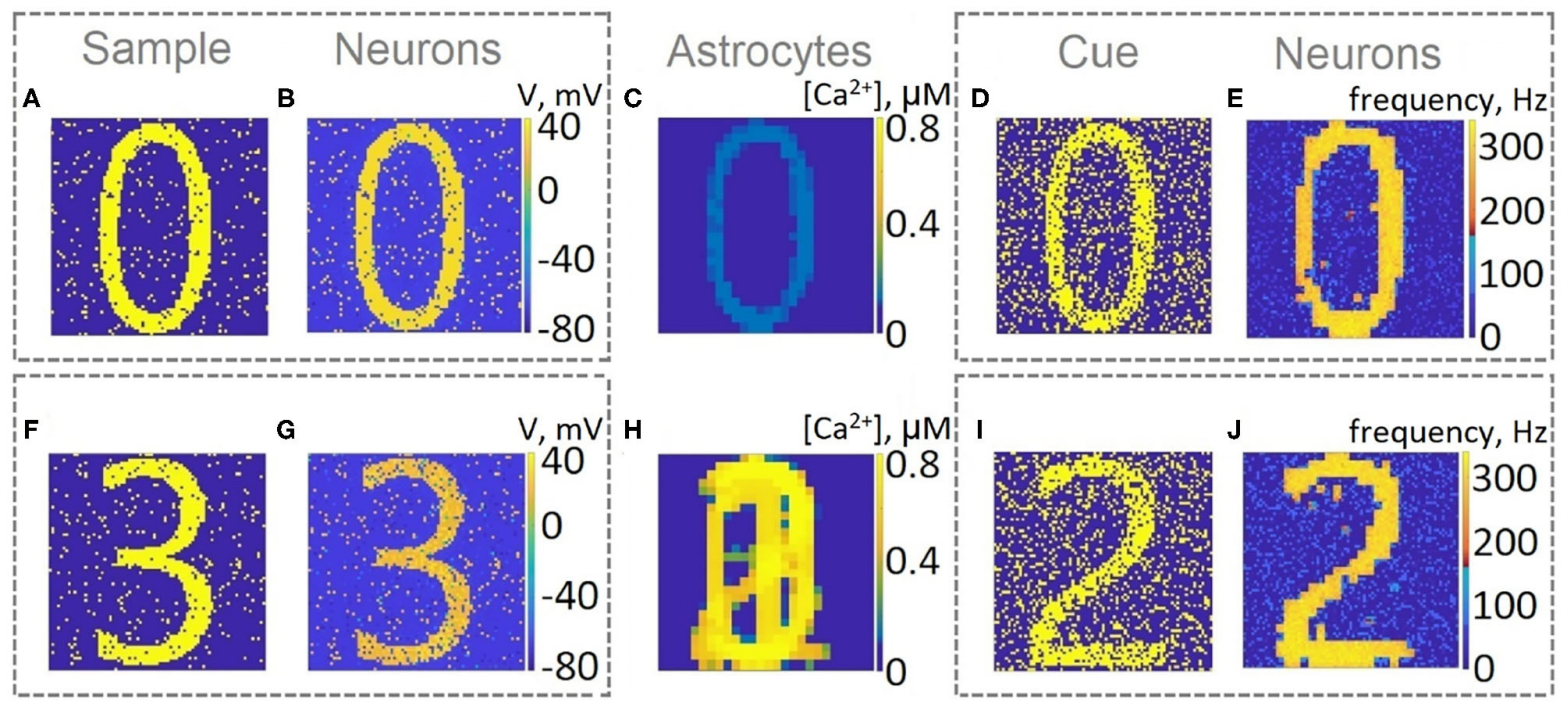
Snapshots of the training **(A,B,C,F,G,H)** and testing **(D,E,I,J)** of the neuron-astrocyte network in the multi-item working memory task. We used the following training set consisting of four samples: 0,1,2,3. **(A,F)** The example of the first and last training samples, respectively. **(B,G)** The response of the neuronal network to samples. The values of the membrane potentials are shown. **(C,H)** The intracellular Ca^2+^ concentrations in the astrocytic layer. **(D,I)** The testing items with 20% salt and pepper noise. **(E,J)** The cued recalls in the neuronal network. The firing rate averaged on the test time interval for each neuron is shown.

Next, we consider the multi-item WM formation. In this case, we loaded four items, images of numerals 0, 1, 2, 3 in the following way. The images were loaded sequentially by applying external inputs of *t*_stim_ durations with 100 ms inter-item intervals (see Figures 2, 6, 7A, 8). Due to the coincidence of different stimulus-specific neuronal populations in space, the spatial calcium patterns in astrocytic layers for different items overlap significantly (Figure 8H). After a 700 ms training stimulus was applied, we tested the maintenance of the memory by applying matching and non-matching items of *t*_test_ durations and 250 ms inter-item intervals (Figures 2, 6, 7A, 8). The images were distorted by 20% noise. The astrocyte-mediated feedback modulating coherent neuronal activity provided the selectivity of the model response. The system remembered the correct image. Thus, we observed that all items were successfully filtered only in the cued recall. Needless to say that firing rate increases significantly in the cued recall due to the selective increase of synaptic strengths (Figures 7B,C). To evaluate the performance of the neuron-astrocyte WM, we used the correlation between recalled item and the ideal item during the multi-item WM task as a metric (see section 3.5; Figure 7D). It is important to note, that during multi-item remembering, spurious correlations never dominate in that sense as accuracy of our system is always equal to 100%. There was an increase in correlation with the target image and no attraction to the wrong image or chimeras. Maximum correlation reached 95% in training and 93% in testing sets on average for four samples. Also note that the model is quite robust to the type of binary images input (images of numerals, letters, etc.). Video of the multi-item WM in the neuron-astrocyte network can be found in Supplementary Material 2.

**Figure 9:**
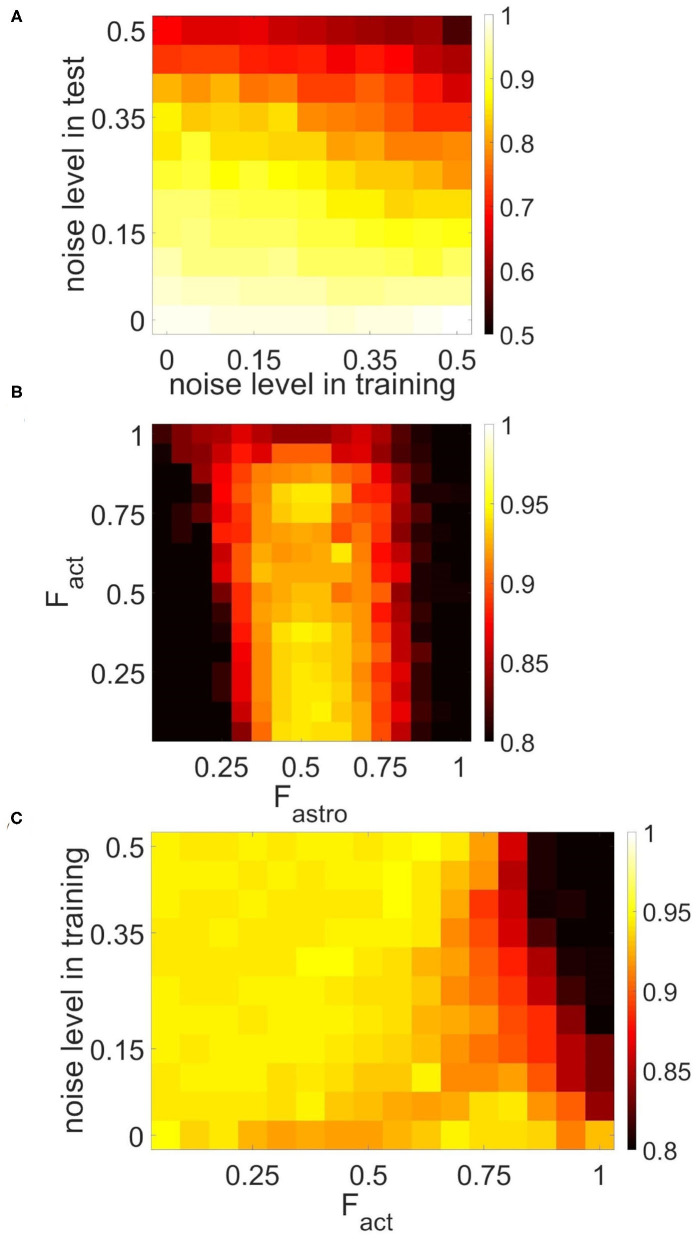
Correlation between recalled pattern (see section 3.5) and ideal item in a multi-item WM task performed by the neuron-astrocyte network. The correlation averaged over four patterns is shown. **(A)** Noise-resistance of the model. Dependence of correlation on the noise level in training and testing. The correlation difference between cued recall pattern and noisy input is shown. **(B,C)** The influence of the neuron-astrocytic interaction structure. **(B)** Dependence of correlation on the number of spiking neurons required for the calcium elevation in the astrocyte, *F*_*act*_, and on the number of spiking neurons required for the emergence of astrocyte-induced enhancement synaptic transmission, *F*_*astro*_. **(C)** Dependence of correlation on noise level in training samples and on the parameter, *F*_*act*_. *F*_*astro*_ = 0.5. For **(B,C)** noise level in cue is 20%.

**Figure 10:**
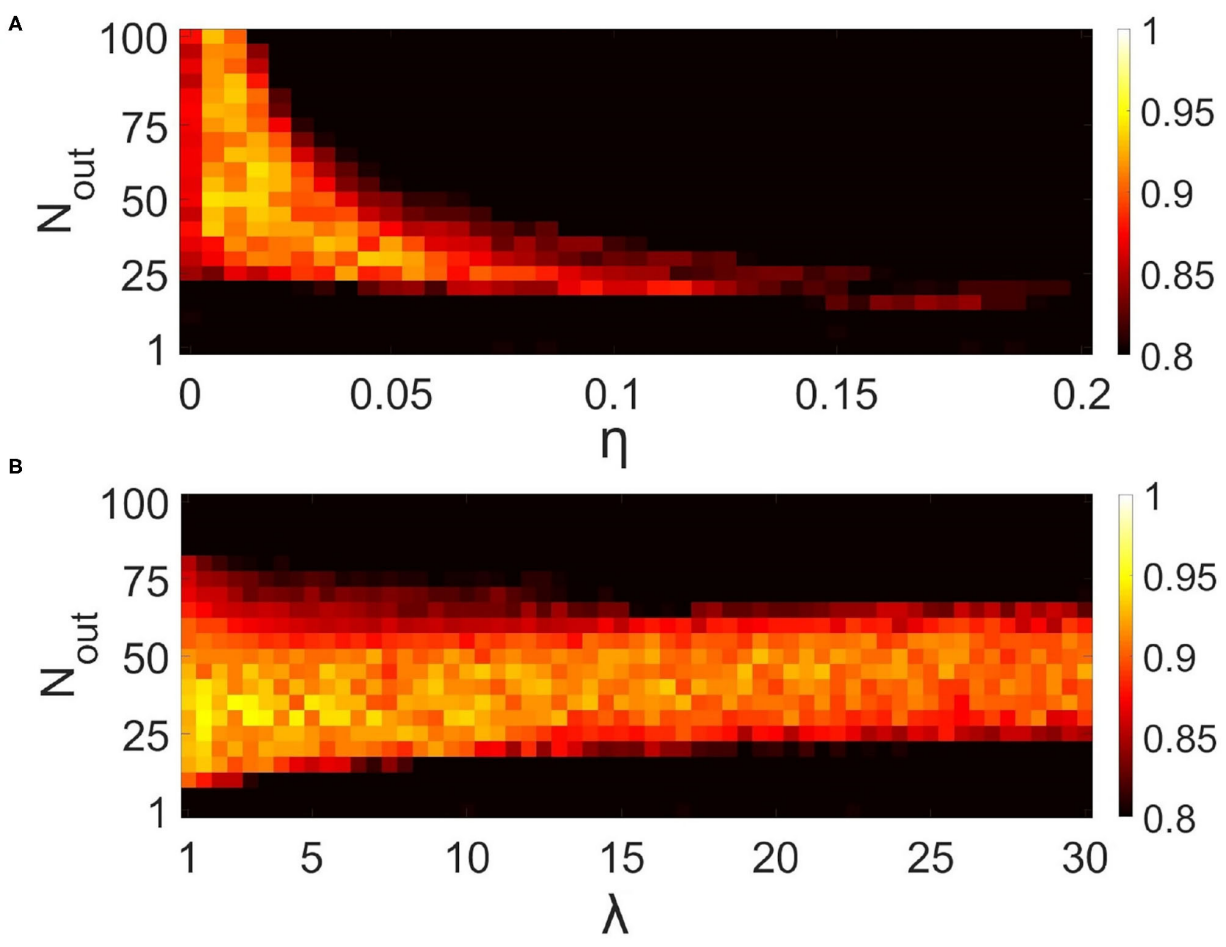
Influence of synaptic connectivities architecture in the neural network on the correlation of recalled pattern in the multi-item WM task performed by the neuron-astrocyte network. The correlation averaged over four patterns is shown. **(A)** Dependence of correlation on the number of output synaptic connections for each neuron, *N*_out_, and synaptic weight, η. λ = 5. **(B)** Dependence of correlation on the number of output synaptic connections for each neuron, *N*_out_, and synaptic connection distribution parameter, λ Equation (4). η = 0.025.

To characterize the quality of memory formation in the model, we examined the dependencies of correlation of retrieval pattern in cued recall on variable parameters of the input patterns and astrocytic, synaptic, and network structure (Figures 9, 10). First, we investigated its dependence on noise parameters. The dependence of correlation of recalled pattern on the noise level in training and test experiments is shown in Figure 9A. Specifically, the correlation difference between the recalled pattern and noisy input is presented. In other words, the model can improve test images depending on noise in training and testing. Training the network with samples with a low noise level (up to 25%) provides a high correlation. The elevation of the noise level in the training sample induces a random activity pattern in the astrocytic network, which in turn leads to noisy recall.

The morpho-functional structure of connections between neurons and astrocytes can affect pattern retrieval in the model (Figures 9B,C). The key parameters determining this structure are the fraction of synchronously spiking neurons of the neuronal ensemble corresponding to the astrocyte required for the emergence of calcium elevation in the astrocyte, *F*_*act*_, and the fraction of synchronously spiking neurons of the neuronal ensemble corresponding to the astrocyte required for the emergence of astrocyte-induced enhancement of synaptic transmission, *F*_*astro*_. Here we do not account for various spatiotemporal properties of gliotransmitter release and astrocyte Ca^2+^ signals evoked by different levels of neuronal activity (Araque et al., 2014). We assume that simultaneous activation of synapses induces multiple Ca^2+^ events at different processes of the astrocyte, which are spatially and temporally integrated and result in the generation of a global long-lasting Ca^2+^ elevation (Bindocci et al., 2017) that can affect synaptic transmission in the territory of individual astrocytes. For this purpose, parameters *F*_*act*_ and *F*_*astro*_ estimate the correlation level of synapse activity in the model. The optimal range of *F*_*astro*_ for correlation of recalled pattern is [0.4–0.6] (Figure 9B). Smaller values of the parameter, *F*_*astro*_, lead to the effect of astrocyte-induced synchronization initiated even by non-stimulus specific uncorrelated noise activity in a small ensemble of neurons. On the contrary, the use of larger *F*_*astro*_ values implies that a highly correlated activity of almost all neurons located in the territory of a given astrocyte is required for the existence of astrocytic modulation of synapses. Hence, the neuron-astrocytic network can not perform a correct recall of noisy cue. Another point is that Figure 9B was obtained for a training set with a low 5% noise level and did not reveal the dependence of the correlation of recalled pattern on the parameter *F*_*act*_. We studied the influence of the parameter *F*_*act*_ on the correlation in the simulations with different noise in training samples (Figure 9C). For lower noise level in training samples, the network memorizes items regardless of the value of the parameter, *F*_*act*_. Increasing the level of noise in the training samples for small values of the parameter, *F*_*act*_, leads to Ca^2+^ elevation in randomly distributed astrocytes first, and then in the whole astrocytic layer. Nevertheless, such non-stimulus-specific astrocytic activation can result in a high correlation of recalled pattern because of the moderate noise level in the cue and optimally chosen value of the parameter *F*_*astro*_. On the contrary, for a larger value of the *F*_*act*_ > 0.85 Ca^2+^ signal in astrocytes can only be evoked by a relatively "clean" sample with a small percentage of noise < 5%, therefore increasing the level of noise in training samples results in poor correlation (Figure 9C). We found that the range of *F*_*act*_ = 0.8 − 0.85 was optimal for performing the WM tasks by the neuron-astrocyte network. In this range, astrocyte activations were stimulus-specific and the astrocyte layer could memorize training samples with a low noise level. The obtained high value of optimal *F*_*act*_ denotes that correct functioning of WM in the neuron-astrocyte network dependent on the generation of global Ca^2+^ signals in astrocytes required highly correlated activity in corresponding neuronal ensembles as confirmed by recent experimental work (Bindocci et al., 2017).

Next, we studied the influence of synaptic connectivity architecture in the neural network, specifically the number, weight, and distribution of synaptic connections, on the correlation in the multi-item WM task (Figure 10). The minimal number of synaptic connections, *N*_out_, required for the existence of cued recall is 20 (Figure 10A). A smaller number of connections is not enough to activate all the neurons from the stimulus-specific population. Simultaneous increase of weights and number of connections induces the generation of large synaptic currents resulting in self-sustained overactivation of the neuron-astrocyte network. Therefore there exists an optimal range of synaptic weight values to ensure high correlation. We found that for our model this range is η ∈ [0.005 − 0.05]. Figure 10B illustrates the dependence of correlation on the number of output synaptic connections and their distribution. The smaller the parameter λ from Equation (4), the lower the probability of long-distance connections. The highest correlation was observed for local connections, λ < 7, due to the fact that short-range connections do not lead to blurring of the pattern retrieval boundaries in the neural network. Figure 10B also determined the optimal range for the number of synaptic connections: *N*_out_ ∈ [25, 55].

The key parameters, which determine WM capacity in the proposed neuron-astrocyte network, are the duration of calcium signals in astrocytes and duration of the astrocyte-induced modulation of synaptic transmission. Duration of astrocytic calcium elevations is determined by the intrinsic mechanisms of the IP_3_-evoked Ca^2+^-induced Ca^2+^ release from the astrocyte endoplasmic reticulum stores, which is described by the biophysical model (Li and Rinzel, 1994) used in this study. Fragmentary experimental data on duration of the gliotransmitter-induced modulation of synaptic transmission shows that the short-lived version of this modulation lasts from fractions of a second to a few minutes, while long-term plasticity can last for tens of minutes (for review see Pittà et al., 2016). In this study we have chosen the duration of astrocytic effect on synaptic transmission in accordance with the kinetics of SICs triggered by astrocytic glutamate (Fellin et al., 2004).

**Figure 11:**
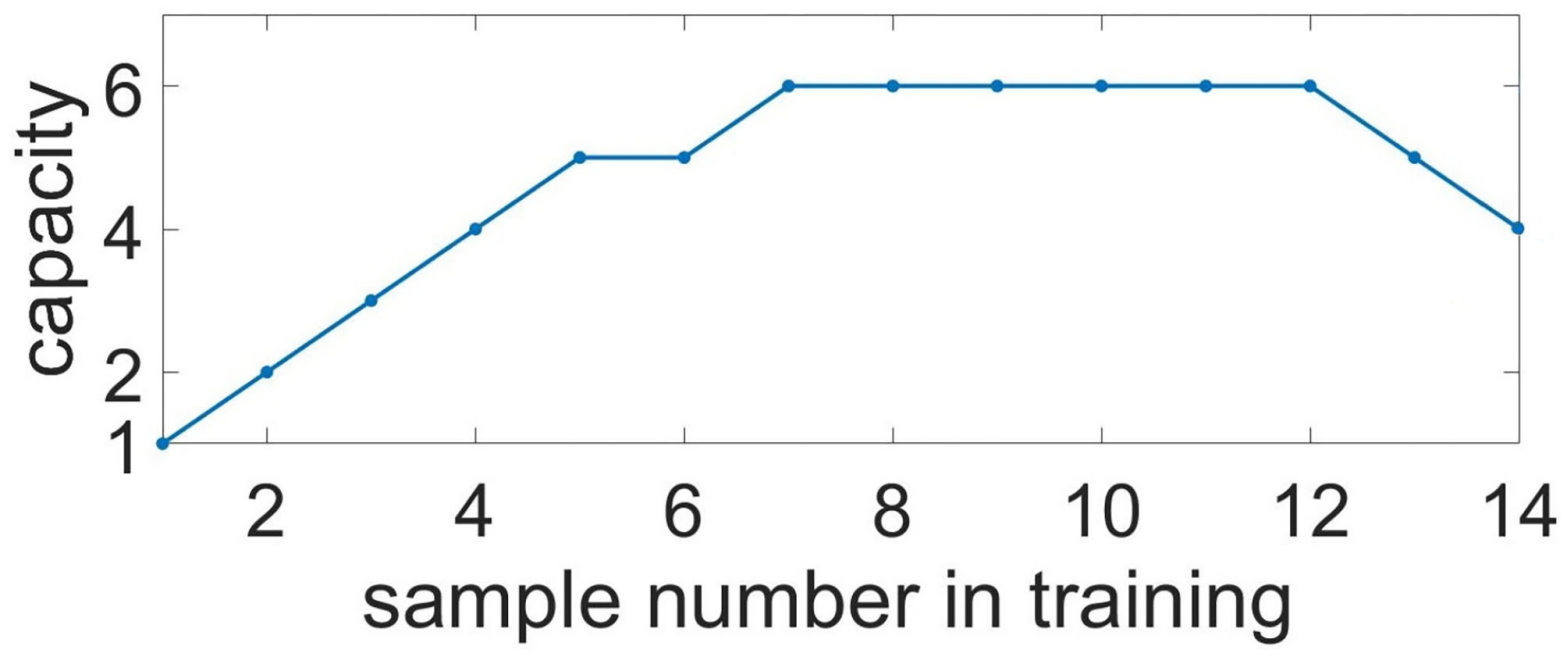
Capacity of the multi-item WM in the neuron-astrocyte network. Capacity as a function of the sample number in training. The number of images with a correlation of recalled pattern higher than 90% is shown.

To characterize memory capacity, we subjected it to longer trains of samples. Samples were applied to neuronal ensembles with an average 35.2% overlapping in population. To check the memorization, we presented cue items in reverse order compared to the learning mode (e.g., learning: 0, 1, 2, ..., 7, 8; test: 8, 7, ..., 2, 1, 0). The number of items with a correlation of recalled pattern higher than 90% indicated the capacity of the system. Figure 11 shows the capacity as a function of the sample number in the training sequence. For a chosen set of parameters, the capacity of WM ranges from five to six. It is interesting that such a limited capacity coincides with psychological studies indicating that human ability to keep information in readily accessible WM is limited, ranging between three and five items for the majority of healthy people (Cowan, 2010). However, it is obvious that the organization of the WM in the human brain involves the coordinated work of a much larger number of cells and even several CNS regions. We investigated the influence of the duration of astrocyte-induced modulation of synaptic transmission, τ_astro_, on the capacity of WM. The number of items stored in the system memory is maximum for parameter values in the range: τ_astro_ ∈ [60, 660] ms. For small values of τ_astro_, excitation does not have time to spread to the stimulus-specific neural population; for long astrocytic modulation the different items in cued recalls interfered with each other.

The capacity of WM in the proposed neuron-astrocyte network is primarily limited by the duration of the astrocytic Ca^2+^ event τ_*Ca*_ and does not depend on the number of neurons or astrocytes in the network. In the protocol of our testing session, the duration of the Ca^2+^ signal is sufficient to keep six samples only, and after that it starts forgetting the images received at the beginning. So, the maximum capacity of the model is six. In case more than 12 items are applied, the network starts dumping them and chimeras appear (Figure 11). So, the system begins to make mistakes and the capacity of the network shrinks (here the capacity is understood as the number of the retrieval items with a correlation level with the sample of more than 90%). This is valid for the case considered in the paper when a stimulus is applied to the whole network and different stimuli overlap strongly in neuronal subnetworks. For this case, the capacity does not depend on the number of neurons and astrocytes.

**Figure 12:**
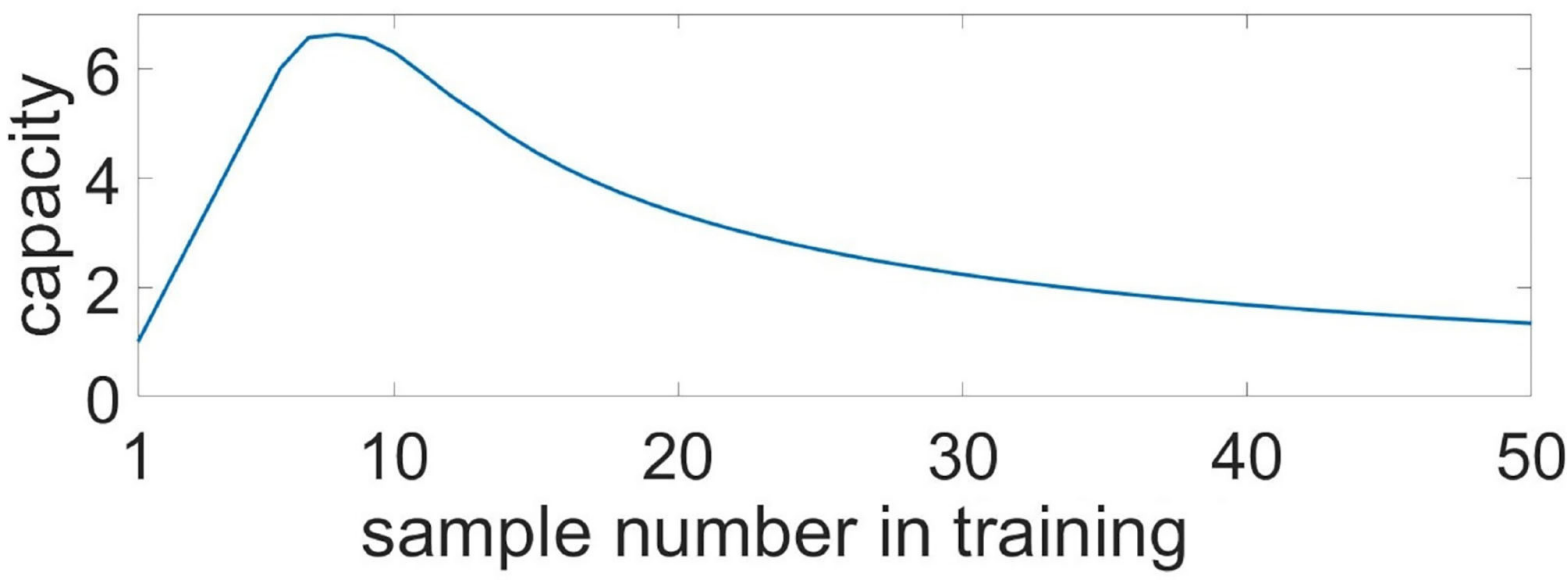
Capacity of the multi-item WM in the neuron-astrocyte network in the case of non-overlapping neuronal populations obtained analytically. Capacity as a function of the sample number in the training sequence.

If we consider the case of applying items to different unique non-overlapping neuronal subpopulations, an increase in the size of the network will result in increased capacity. The WM capacity of each subnetwork in this case is unequivocally determined by the duration of the calcium signal and can be obtained analytically. We estimated the Ca^2+^ signal duration to be τ_*Ca*_ = 3.8 s. We consider the case of training on *K* samples in a fixed order: 1, 2, ..., *K*. During the test, we look at a permutation *p* consisting of *K* patterns. For the permutation, we estimate the number of correctly recalled patterns, *K*_*p*_. The pattern is considered correctly recalled if no more than τ_astro_ has passed since its presentation. The average capacity, *C*, for this case is defined as the average number of correctly recalled patterns over all possible permutations *p*. According to this description, the average capacity can be calculated by the following equation:

(17) $$\begin{array}{l} \begin{array}{l} t_{train}^{i}=i\cdot \tau_{11}+\left(i-1\right)\tau_{12},\\ t_{test}^{j,i}=t_{train}^{i}+\tau_{shift}+j\cdot \tau_{21}+\left(j-1\right)\tau_{22},\\ C=\frac{1}{K!}\underset{p}{\sum }\overset{K}{\underset{1}{\sum }}\left[t_{test}^{p_{i},i}-t_{train}^{i}<\tau_{Ca}\right]=\\ \frac{1}{K!}\overset{K}{\underset{1}{\sum }}\underset{p}{\sum }\left[t_{test}^{p_{i},i}-t_{train}^{i}<\tau_{Ca}\right]=\\ \frac{\left(K-1\right)!}{K!}\overset{K}{\underset{1}{\sum }}\overset{K}{\underset{j=1}{\sum }}\left[t_{test}^{j,i}-t_{train}^{i}<\tau_{Ca}\right], \end{array} \end{array}$$

where τ_11_—train sample duration, τ_12_—duration between train samples, τ_21_—test sample duration, τ_22_—duration between test samples, τ_*shift*_—delay between train and test, τ_*Ca*_—calcium event duration, $t_{train}^{i}$—time of i-th train sample finishes, $t_{test}^{j,i}$. Figure 12 shows the capacity as a function of the sample number, *K*. After it reaches a maximum of 6.6 for 8 samples, the capacity begins to decrease monotonically, while the number of samples increases.

## 5. Discussion

We proposed a new biologically motivated spiking neuron network model accompanied by astrocytes that demonstrates working memory formation. The model acts at multiple timescales: at a millisecond scale of firing neurons and the second scale of calcium dynamics in astrocytes. The neuronal network consists of randomly sparsely connected excitatory spiking neurons with non-plastic synapses. Astrocyte-induced activity-dependent short-term synaptic plasticity results in local spatial synchronization in neuronal ensembles. The WM realized by such astrocytic modulation is characterized by one-shot learning and is maintained for seconds. The astrocyte influence on the synaptic connections during the elevation of calcium concentration implements Hebbian-like synaptic plasticity differentiating between specific and non-specific activations. Note that the proposed model is crucially different from the attractor-based network memory models (Hopfield, 1982; Amit, 1995; Wang, 2001; Wimmer et al., 2014) and works similarly to WM models based on synaptic plasticity (Mongillo et al., 2008; Lundqvist et al., 2011; Mi et al., 2017; Manohar et al., 2019). In particular, in its functionality, the model is quite close to short-term associative (Hebbian) synaptic facilitation (Sandberg et al., 2003; Fiebig and Lansner, 2016).

**Figure 13:**
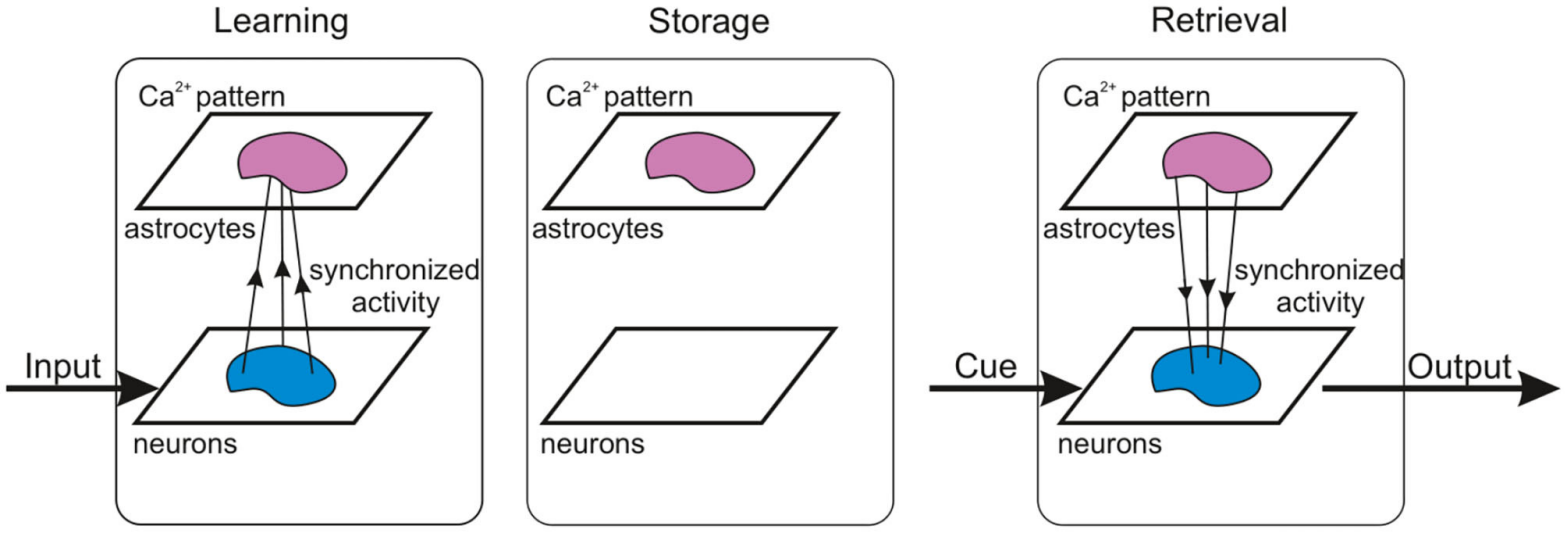
Concept of WM operation in the spiking neuron network model accompanied by astrocytes.

The concept of our WM model operation is schematically summarized in Figure 13. Composed of two building blocks, e.g., fast-spiking neurons and slow astrocytes, the proposed memory architecture eventually demonstrated synergetic functionality in loading information and its readout by the neuronal block and storage implemented by the astrocytes. In contrast with solely neuronal circuit models where memory is encoded in synaptic connections and their plasticity, which inevitably leads to the problem of overlapping, our model splits functionality using astrocytes as a pool for stored patterns. Even with significant overlaps, they can be successfully retrieved due to coherent synaptic modulations by the astrocytes and synchronous neuron firing, which provide the selectivity. When the memory is maintained at the time scale of calcium elevation in astrocytes, the synapses are not specifically modulated and theoretically can be employed for other tasks. Note also that the memory is transient with no long-lasting changes in structure and parameters. Thus, new information can be successfully uploaded and stored without interference of traces of previously memorized information. Complete memory overwrite interval is estimated at several seconds and is also defined by the duration of astrocyte activations. The circuit in Figure 13 can be also treated as a building block for functional spiking neuron networks (SNN) which are now intensively discussed in the IT community as an alternative to artificial neural networks (ANNs) used in machine learning and artificial intelligence. Theoretically, SNNs as more accurate models of brain circuits are believed to be much more powerful than traditional formal networks in processing efficiency. However, learning and memory algorithms working well in ANNs can not be directly translated to spiking neurons representing, in fact, analogous dynamical systems. We hypothesized that astrocytes may be a missing block in the implementation of learning and memory in SNNs as systems to solve information processing tasks.

The proposed model clearly confirms the theoretical hypothesis that astrocytic modulation of synaptic transmission can be involved in the formation of functional cortical WM. We show that the potentiation of excitatory synapses induced by the glutamate released from astrocytes could serve as a possible molecular mechanism for WM. Stages of multi-item WM, which include loading, storage, and cued recall, manifest in brief oscillatory bursts, which are functionally similar to WM activity in nonhuman primate PFC (Lundqvist et al., 2016) rather than sustained neuron spiking. Interestingly, the activity of the neuron-astrocyte network corresponding to a memorized pattern exhibits a sufficient degree of stability, which ensures memory retention despite the presence of significant overlaps in the stimulus-specific subnetworks.

Needless to say the astrocyte-induced modulation of synaptic transmission proposed in this study as a mechanism for WM organization does not exclude but rather complements other synaptic and neural plasticity mechanisms (fast Hebbian synaptic plasticity/short-term synaptic plasticity, facilitation, augmentation, dendritic voltage bistability, etc.) and may well act in parallel to them.

On the one hand, there has been much experimental evidence that astrocytes contribute to synaptic plasticity, coordination of neural network oscillatory activity, and memory function (Santello et al., 2019). It was shown recently that astrocytic impact is circuit-specific (Martin et al., 2015) and stimulus-specific (Mariotti et al., 2018). Improved Ca^2+^ imaging approaches have identified a spatiotemporal diversity of astrocytic signals that may underlie the capacity of astrocytes to encode and process different patterns of activation (Bindocci et al., 2017; Stobart et al., 2018). Besides, the temporal scale of the astrocytic calcium dynamics and dynamics of the neuron-astrocyte bidirectional communication including the effects of astrocytic influence on synaptic plasticity fit very well in the timing required in WM processes.

On the other hand, the ongoing intense debate about principles of WM organization challenges the canonical theory of persistent delay activity in network attractors with recurrent excitation (Bouchacourt and Buschman, 2019) and offers alternative models incorporating different biophysical network mechanisms of WM (Barak and Tsodyks, 2014; Lundqvist et al., 2018). The principal reasons for such a debate are the reexamination of experimental data, which show a large heterogeneity in delay neuronal activity during WM tasks (Stokes et al., 2013). A non-classical WM model includes short-term synaptic plasticity (Mongillo et al., 2008; Hansel and Mato, 2013), the balance of inhibition and excitation (Boerlin et al., 2013), NMDA currents affecting on the neuronal excitability (Durstewitz, 2009), and other parameters. These models, however, have a number of shortcomings: inability to describe encoding of novel associations in synaptic facilitation-based models; unclear mechanisms for achieving precise tuning of recurrent excitation and inhibition; and the time constant of the NMDA receptor is appropriate to maintain memories for 1–5 s, but not for longer. The investigation of the synaptic mechanisms underlying WM is an ongoing process. Therefore, incorporation of astrocytes as spatiotemporal integrators and modulators of synaptic transmission in neural network models may help advance the theoretical framework of WM encoding and maintenance mechanisms.

Our simulations in a biologically relevant but still quite general model have eventually illustrated the hypothesis of astrocytes participation in WM functioning. This hypothesis emerged from several experimental facts on the astrocyte contributions to the cognitive functions and their impairments (Santello et al., 2019; Gordleeva et al., 2020; Whitwell et al., 2020). Indeed, in many of these cases, the understanding of the precise mechanisms of the astrocytic involvement is quite fragmentary and more work on the specific role of astrocytic action in the memory processes is needed. We believe that our model demonstrating WM functionality based on a quite clear mechanism of inter-coordination between fast spiking neuronal circuits and an astrocyte reservoir will be helpful in further experimental research including cellular and *in vivo* studies. In particular, depressing or facilitating the neuron-astrocyte interaction by specific drug injections can be used to monitor behavior impairments in animal studies.

To conclude, the recent controversies in the field of WM and the constant growth of experimental evidence about the participation of astrocytes in information processing, cognitive function, and dysfunction open up questions about mechanisms of astrocyte involvement in memory formation. Using a computational model, we demonstrated that the astrocyte-induced facilitation of excitatory transmission in PFC is a plausible mechanism for WM organization. The proposed model accounts for the astrocytic modulation of synaptic transmission in a spiking neural network. The biologically relevant neuron-astrocytic network implements loading, storage, and cued retrieval of multiple items presented to the neuronal populations with significant overlapping. The mechanism of WM functioning in the model is based on coherent neuronal firing in response to sensory inputs which are coordinated by astrocytes in time, serving as a “memory reservoir” and in space serving as a “signal bridge” providing a certain level of synchrony.

Future research in the framework of the proposed WM model in the neuron-astrocyte network will be focused on the interplay of excitation and inhibition that can stabilize WM (Boerlin et al., 2013); the effects of synaptic plasticity (Mongillo et al., 2008; Hansel and Mato, 2013) namely associative short-term potentiation (a fast-expressing form of Hebbian synaptic plasticity) that can provide an encoding of novel associations (Fiebig and Lansner, 2016); the subcellular calcium dynamics in astrocytes (Bindocci et al., 2017; Gordleeva et al., 2019); and on the structure of the cortical microcircuit reflecting the columnar organization of the neocortex.

## Data Availability Statement

The raw data supporting the conclusions of this article will be made available by the authors, without undue reservation.

## Author Contributions

All authors jointly worked on all aspects of the study and the preparation of the manuscript. The literature analysis, coding, and data gathering were mainly done by YT, MK, SG. The manuscript was mainly written by SG and VK. AZ, MI and AG supervised the work.

## Conflict of Interest

The authors declare that the research was conducted in the absence of any commercial or financial relationships that could be construed as a potential conflict of interest.
